# Automatic Screening of Diabetic Retinopathy Using Fundus Images and Machine Learning Algorithms

**DOI:** 10.3390/diagnostics12092262

**Published:** 2022-09-19

**Authors:** K. K. Mujeeb Rahman, Mohamed Nasor, Ahmed Imran

**Affiliations:** College of Engineering & Information Technology, Ajman University, Ajman P.O. Box 346, United Arab Emirates

**Keywords:** diabetic retinopathy, machine learning, GLCM feature, fundus image, image segmentation, MATLAB, SVM, DNN

## Abstract

Diabetic Retinopathy is a vision impairment caused by blood vessel degeneration in the retina. It is becoming more widespread as it is linked to diabetes. Diabetic retinopathy can lead to blindness. Early detection of diabetic retinopathy by an ophthalmologist can help avoid vision loss and other complications. Diabetic retinopathy is currently diagnosed by visually recognizing irregularities on fundus pictures. This procedure, however, necessitates the use of ophthalmic imaging technologies to acquire fundus images as well as a detailed visual analysis of the stored photos, resulting in a costly and time-consuming diagnosis. The fundamental goal of this project is to create an easy-to-use machine learning model tool that can accurately predict diabetic retinopathy using pre-recorded digital fundus images. To create the suggested classifier model, we gathered annotated fundus images from publicly accessible data repositories and used two machine learning methods, support vector machine (SVM) and deep neural network (DNN). On test data, the proposed SVM model had a mean area under the receiver operating characteristic curve (AUC) of 97.11%, whereas the DNN model had a mean AUC of 99.15%.

## 1. Introduction

Diabetes is a long-term but controllable disease that happens when the pancreas does not make enough insulin or when the body does not use the insulin it makes. Insulin is a hormone that regulates the amount of sugar in the bloodstream. Over time, physiological systems are harmed by excessively high blood sugar levels. Diabetes can cause long-term complications such as diabetic nephropathy (kidney disease), strokes and heart attacks, neuropathy (nerve damage), diabetic foot ulcers, and diabetic retinopathy (DR) [1]. DR is a condition that causes damage to the retina. The retina is a light-sensitive layer on the eye’s backside. When blood vessels in the retina are destroyed, it results in DR, which might cause minor vision problems at first but eventually leads to blindness. Non-proliferative diabetic retinopathy (NPDR) is the first stage of diabetic retinopathy, in which microscopic bulges known as microaneurysms grow in the retina’s tiny blood vessels, allowing blood to seep into the retina [2,3,4]. DR is the main cause of eyesight loss in working-age individuals (20–65 years), according to the International Diabetes Federation (IDF). One in every three diabetics is affected by DR, and one in every ten positive cases develops a vision-threatening form of the disease. The IDF has announced that 537 million people worldwide have diabetes, up 16 percent (74 million) from previous IDF forecasts for 2019 [5,6]. Because DR is becoming more common, it is projected to be one of the leading causes of vision impairment or blindness in the future. As a result, an eye exam is an important element of diabetes care. The earlier DR is discovered, the more quickly it can be treated. As a result, individuals diagnosed with diabetes should see an ophthalmologist right away for medical treatment to prevent blindness.

Machine learning (ML) and artificial intelligence (AI) have successfully solved complicated issues in almost every area in recent years, as proven by the scientific community. The importance of ML and AI in medicine is growing [7,8,9]. In the future, ML and AI will be used to improve patient care by personalizing medication and adapting it to the needs of individual patients. We employed ML algorithms to estimate DR using retinal scans in this study. The key features of the fundus images were identified using a grey-level co-occurrence matrix (GLCM). We created two ML classifier models, SVM and DNN, that make use of GLCM characteristics extracted from fundus images, and we conducted multiple tests to identify the most accurate DR classifier for categorizing any unknown retinal scan into healthy or DR.

The following sections make up the remainder of this paper: Section 2 offers background and a review of recently published research papers. Section 3 details the materials and methods employed in the research. The findings of numerous trials are presented in Section 4, which is followed by the conclusion in Section 5.

**Figure 1:**
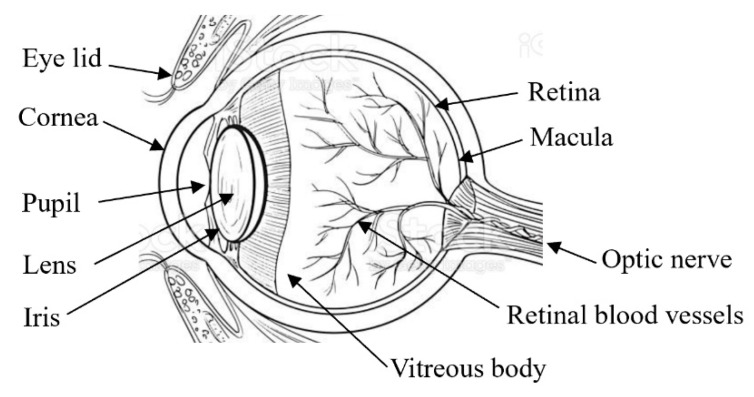
Anatomy of the eye.

The eye is a sensitive organ and a crucial part of the visual system. It is a spherical hollow sphere with an uneven shape. It has the incredible capacity to absorb light rays reflected from physical objects in our environment and convert them to corresponding images [10]. Figure 1 presents the anatomy of the eye with its vital components [11]. The cornea is a clear domelike structure placed in front of the eye covering the iris or colored part of the eye, which controls the amount of light entering it. The lens is the transparent region of the eye beneath the iris that concentrates incident light on the retina. The retina processes light into electrical impulses that pass via the optic nerve (located on the back of the eye) and are transmitted to the brain for further processing [12].

Diabetic patients are prone to several complications, one of which is DR. Diabetic people, particularly those with uncontrollably high blood sugars, are susceptible to this eye disease [13]. DR is a consequence of diabetes that damages the blood vessels at the back of the eye and may lead to blindness if left misdiagnosed and untreated [3,4,5]. The variants of DR are proliferative retinopathy and macular edema. The former is caused by blood flow into the center of the eye from damaged vessels, causing blurred vision. The latter is a more advanced situation in which the fluid leaks into the macula’s center and leads to partial or complete blindness [3,4,5]. A comprehensive eye exam that includes the following tests can detect macular edema and diabetic retinopathy: A visual acuity test that measures how well you see at various distances, followed by a dilated eye test, a type of eye exam in which certain drops enlarge the pupils, allowing the eye specialist to examine the retina and optic nerve for signs of damage with the help of a magnifying lens. The specialist may also conduct a tonometry test to measure the pressure inside the eye using special instruments [14]. The presence of hard exudates, microscopic white or yellowish-white coatings with sharp edges in the retina’s outer layers, is the most apparent symptom of DR [13]. These hard exudates are visible on the retinal scans, making it easy for an expert to diagnose the condition. Many different retinal/fundal imaging devices currently in use can provide high-quality digital images of the retina.

Many research articles have been published on detecting DR utilizing fundus images and ML methods in recent years [15,16,17,18,19]. In some investigations, the researchers used binary fundus image datasets, and the rest used multiclass datasets. As learned from the research publications, the three critical processes involved in the DR prediction algorithms are preprocessing, feature extraction, and classification. Different image enhancement approaches have been used to preprocess the input images. Convolutional Neural Network (CNN) and Gray Level Co-occurrence Matrix (GLCM), and Local Binary Patterns (LBP) are some of the most commonly used feature extraction techniques. Most recent papers employed CNN as feature extraction. This study uses binary datasets with retinal images divided into healthy and DR groups.

Ahmad Z. et al. (2018) created a DR classifier using the GLCM as a feature extractor and an SVM with four kernel functions (quadratic, linear, Gaussian, and polynomial) as a binary classifier. The accuracy scores of the model for the four kernels were: 72.72% (quadratic), 22.72% (linear), 63.64% (gaussian), and 90.91% (polynomial). The results indicate that the polynomial kernel function looks to be more suitable for DR classification [15].

Using a CNN model, K. Xu et al. (2019) automatically classified 1000 retina images in the Kaggle dataset as healthy or DR images. Before feeding the images to the CNN, the images were scaled to a size of 224 × 224 × 3. Additionally, the authors applied image augmentation techniques to populate the original dataset. Eight Conv layers, four max-pooling layers, and two FC layers made up the CNN architecture. For classification, the SoftMax function was applied to the final layer of CNN. The accuracy of this approach was 94.5% [16].

E. Dhiravidachelvi et al. (2019) introduced an approach for detecting and classifying microaneurysms in diabetic retinopathy fundus scans. Initially, a median filter is applied to the images, followed by contrast-constrained adaptive histogram equalization (CLAHE) as a preprocessing step. The authors then used the GLCM algorithm to extract features before feeding them to a k-nearest neighbor (KNN) binary classifier. On the test datasets, the model had a maximum accuracy of 93%. The model had maximum sensitivity and specificity of 95.7% and 90.56%, respectively. According to the researchers, the proposed method could be used to detect a variety of pathologies in retinal images of varying quality levels in the future [17].

Elveny M. et al. (2020) introduced a probabilistic neural network (PNN) ML classifier to identify diabetes retinopathy using fundus pictures. The raw images were first preprocessed using operations such as resizing, getting green channel, and contrast stretching in this work. To extract image features, the researchers applied GLCM in the second stage. Finally, to create the predictions, the probabilistic neural network was used. According to the test findings, the approach identified diabetic retinopathy with an accuracy of 86.8 % [18].

Ramzi A. et al. (2021) suggested an ML technique for identifying and categorizing DR. The authors adopted local binary patterns (LBP) as a feature extractor and investigated the performance of several state-of-the-art pre-trained deep learning models for classification. The best three models, ResNet, DenseNet, and DetNet were found to have accuracies of 96.35%, 84.05%, and 93.99%, respectively. The ResNet model took 34 min on an NVIDIA^®^ GeForce GTX 1050Ti with a memory 4 GB (NVIDIA Corporate, 2788 San Tomas Expressway, Santa Clara, CA 95051) [19].

We observed from the above research articles that the datasets used in these studies were not unique; they differed in terms of the number of images, resolution, and the devices used to capture the images. In addition, we learned that the researchers tried out different types of feature extraction techniques, such as GLCM, LBP, and Resnet (CNN); however, the most explored feature extraction technique was the GLCM. To build the DR classifier, the researchers adopted traditional ML algorithms such as SVM and KNN and neural network models such as PNN and DNN. We noticed that the highest accuracy using SVM and KNN algorithms was 90.91% and 93%, respectively. The KNN model that used GLCM had a maximum sensitivity and specificity of 95.7% and 90.56%, respectively. The ResNet model based on LBP features achieved 96.35% accuracy. 

The following are some of the limitations of the research studies mentioned above:

(a) Many researchers used accuracy as the only metric to evaluate the performance of their models. However, sensitivity and specificity are essential when examining how well ML classifier models work.
(b) The state-of-the-art model, the ResNet, has a complex structure and has to calculate millions of parameters while being trained. This means that a powerful computer (GPU) is needed to build the model.
(c) Most authors did not cross-validate the model’s test scores; instead, they used maximum accuracy scores based on fixed test samples, which may not be realistic.

Therefore, the main goal our research is to create lightweight ML models that use GLCM features of fundus images to accurately classify DR with higher accuracy, sensitivity, and specificity. To accomplish the stated objectives, we adopted relatively less complex but efficient fundus image segmentation algorithm and extracted ten salient GLCM features (as compared to five features in the relevant studies). We also experimented using a SVM and a DNN to find the best performing DR classifier model.

## 2. Materials and Methods

### 2.1. Data

**Figure 2:**
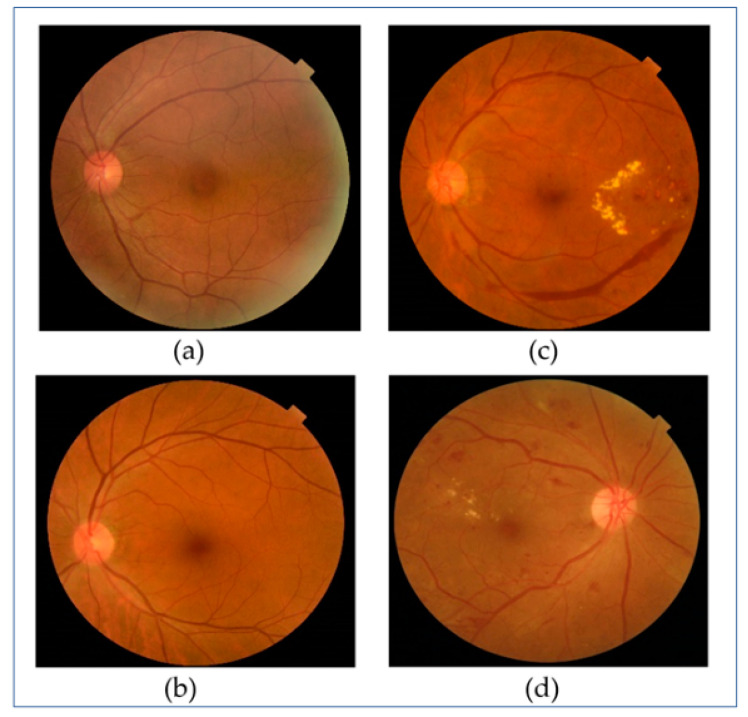
Sample images from both classes: (**a**,**b**) Healthy fundus. (**c**,**d**) DR fundus.

To create a dataset for the proposed DR classifier, we acquired pre-recorded normal and retinopathic fundus images from four reliable public data repositories: Kaggle [20], DDR [21], Zenodo [21], and Mendeley [22]. Table 1 outlines the key characteristics of the images used in our investigation. There are 560 images in this collection, separated into two categories (normal and DR), each with 280 images. Figure 2 depicts two image examples from each of the two classes.

### 2.2. Methodology

**Figure 3:**
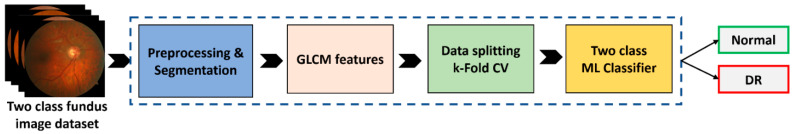
An overview of the proposed DR classifier.

An overview of the proposed DR classifier is depicted in Figure 3. The key steps include preprocessing, segmentation, GLCM feature extraction, data splitting, and making predictions using a two class ML classifier. The following sections give a quick overview of each operation.

#### 2.2.1. Preprocessing and Segmentation

**Figure 4:**
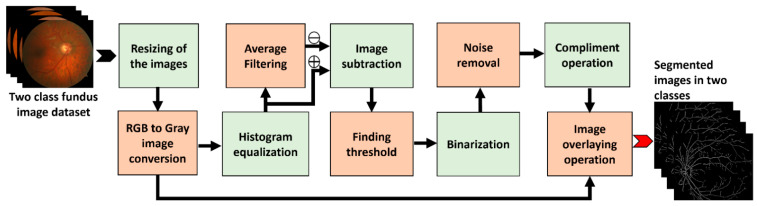
Overview of the proposed DR classifier.

This step is used to improve the overall quality of the fundus images in the datasets and to separate the different parts of the fundus, such as blood vessels, exudates, and microaneurysms (MAs). Figure 4 shows the step-by-step procedures for the pre-processing and segmentation. 

The operation starts with the fundus photos being loaded from an image data folder. As indicated in Table 1, the size of the fundus images in the data folder is different, so the next step is to resize the images. A smaller image size allows ML algorithms to run faster; therefore, experimentally, we found the optimal size at 512 × 512. In the next stage, the resized images in RGB format are converted to grayscale images, as the grayscale images retain the most relevant information associated with DR; the resulting reduction of one-third in the image size further speeds up the computation. To enhance the overall quality of the image, we then applied histogram equalization to the grayscale images [23,24]. We then used an averaging filter mask of size 9 × 9 (consisting of all 1 s) to find the average information and subtract it from the histogram-equalized image. This step removes all the background information from histogram equalized images and only keeps high-frequency components, such as blood vessels, exudates, or MAs (if present). We then calculated the global image threshold using the iterative method recommended by Ridler and Calvard [25]. This threshold can be used to transform a grayscale image into a binary image. The first step in the thresholding stage is to normalize the intensity value of the input image to fall between 0 and 1. The histogram is initially divided into two halves using a starting threshold value and half the maximum dynamic range. The sample mean of the intensity values related to the foreground and background pixels is then calculated. These two sample averages are used to calculate a new threshold value. Until the threshold value becomes constant, the process is repeated depending on the new threshold. Binary images are then derived from the subtracted image using the above threshold, and the pixel below the threshold forms black dots; if it is equal to or above the threshold, it forms the white dots of the resulting binary image. In the next step, we reduce the noise from the binary image by discarding the pixel clusters with 50 or fewer white pixels (we fix this value using several rounds of experimental trials). The next step was a complement operation, which converts black to white and vice versa, such that all the blood vessels appear black with a white background. In the last stage, segmentation DR images are realized by overlaying an inverted image on the grayscale image. As a result, the output image only contains blood vessels, exudates, and MAs.

#### 2.2.2. GLCM Feature Extraction

**Figure 5:**
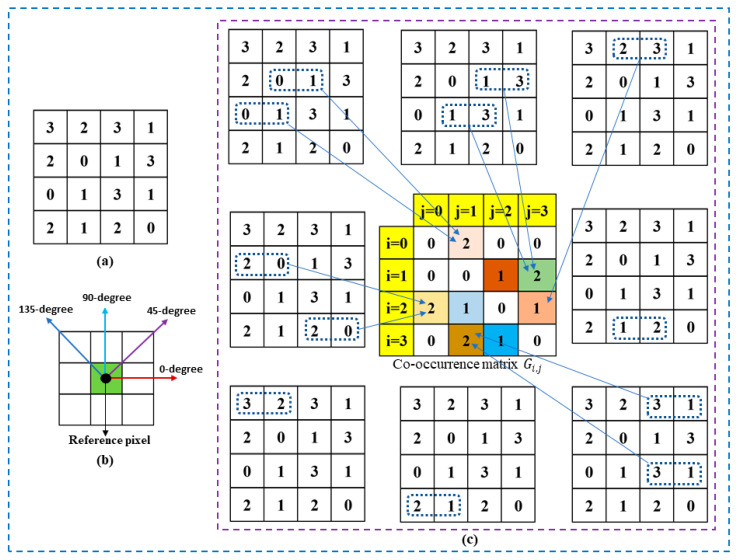
Computation of GLCM from an image patch: (**a**) The input image patch; (**b**) A reference pixel in the given image and the preferred orientations of GLCM; (**c**) Computation of the co-occurrence matrix. Note: Different colors are used in the above diagram to indicate specific pixel pair combinations.

We can adopt multiple methods to perform texture analysis of digital images, but the most common is GLCM. The GLCM can be used to compare the difference in gray levels between any two pixels next to each other in any given direction of an image. The GLCM is a square matrix representing the frequency of specified pairings of gray levels, G(i, j) co-occurring in a given image or an image segment horizontally, vertically, or diagonally. In texture feature calculations, the GLCM matrix is used to figure out how the intensity changes at the pixel of interest [26,27]. Figure 4 illustrates the computation of GLCM for a given image. To best explain the GLCM algorithm, we consider an image patch of size 9 × 9 shown in Figure 5a, which consists of gray levels 0 to 3. The typical GLCM directions for any reference pixel are indicated in Figure 5b. As shown in Figure 5c, the GLCM can be found by figuring out how often pairs of pixels in the image {(3, 2), (2, 3), (3, 1), (2, 0), (0, 1), (1, 3), (2, 1), (1, 2)} appear together. For convenience, we then divide each GLCM element by the sum of all GLCM elements to get a normalized GLCM with elements in the range 0 to 1.

In the proposed methodology, the GLCM algorithm takes the segmented gray level images as input and outputs the following statistical features [26,27,28].

(a) Contrast

The contrast of an image is defined as the difference between the highest and lowest values of a pixel. This feature provides how much local variation there is in the picture. Equation (1) can be used to calculate the contrast of an image, where $G_{i,j}$ is an element of the given GLCM matrix at (*i*, *j*), *N* number of gray levels in the segmented gray level image.

(1) $$Contrast=\overset{N-1}{\underset{i,\;j=0}{\sum }}G_{i,j}{\left(i-j\right)}^{2}$$

(b) Energy

Energy is a statistical measure used to quantify texture uniformity. An image that has a steady gray levels or periodic pattern provides high energy. Equation (2) defines energy.

(2) $$Energy=\overset{N-1}{\underset{i,\;j=0}{\sum }}G_{ij}^{2}$$

(c) Entropy

Entropy is a measure of randomness in the distribution of intensity levels in an image. This statistic can be used to understand the texture of an image. Entropy can be found using Equation (3).

(3) $$Entropy=-\overset{N-1}{\underset{i,\;j=0}{\sum }}G_{i,j}\ln G_{i,\;j}$$

(d) Homogeneity

The local gray level homogeneity is determined by this metric. It has the maximum value when all elements in an image are the same, indicating that the image is highly homogeneous. Homogeneity can be calculated using Equation (4).

(4) $$Homogeneity=\overset{N-1}{\underset{i,\;j=0}{\sum }}\frac{G_{i,j}}{1+{\left(i-j\right)}^{2}}$$

(e) Correlation

Correlation is a statistical measure that gives pixel pair connection or reliance in an image. Equation (5) shows the correlation equation, where μ and σ denotes mean and standard deviations of GLCM matrix, respectively, and can be estimated using Equations (6)–(9).

(5) $$Correlation=\overset{N-1}{\underset{i,\;j=0}{\sum }}G_{i,j}\;\frac{\left(i-\mu_{i}\right)\left(j-\mu_{j}\right)}{\;\sigma_{i}\sigma_{j}}$$

(6) $$\mu_{i}=\overset{N-1}{\underset{i,\;j=0}{\sum }}i\;G_{i,j}$$

(7) $$\mu_{j}=\overset{N-1}{\underset{i,\;j=0}{\sum }}j\;G_{i,j}$$

(8) $$\sigma_{i}=\sqrt{\overset{N-1}{\underset{i,\;j=0}{\sum }}G_{i,\;j}{\left(i-\mu_{i}\right)}^{2}}$$

(9) $$\sigma_{j}=\sqrt{\overset{N-1}{\underset{i,\;j=0}{\sum }}G_{i,\;j}{\left(j-\mu_{j}\right)}^{2}}$$

(f) RMS

The RMS is the root mean square value of each input row or column, along with vectors of a chosen input dimension, or for the whole input. Equation (10) may be used to compute RMS.

(10) $$RMS=\sqrt{\frac{1}{\left(N-1\right)}\overset{N-1}{\underset{i,j=0}{\sum }}G_{i,j}{}^{2}}$$

(g) Skewness

The degree of asymmetry in the pixel distribution in the specified window around its mean is referred to as skewness. Skewness is a single metric that describes the shape of a distribution. The following is the formula for determining skewness:

(11) $$RMS=\sqrt{\frac{1}{\left(N-1\right)}\overset{N-1}{\underset{i,j=0}{\sum }}G_{i,j}{}^{2}}$$

(h) Kurtosis

Kurtosis measures how stable a distribution is in comparison to a normal distribution.

(12) $$Kurtosis=\frac{1}{\left(N-1\right)\sigma^{4}}\overset{N-1}{\underset{i,j=0}{\sum }}{\left(G_{i,j}-\mu \right)}^{4}$$

#### 2.2.3. Data Splitting by K-Fold CV

**Figure 6:**
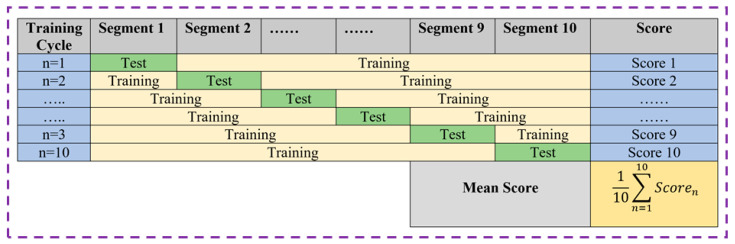
Data splitting by k -fold cv.

One of the most important steps in constructing ML models is organizing data into a training set and a test set. The training set is used to train an empty model, whereas the test set is used to evaluate the correctness of the model. The random 70:30 method is the most common data split method, in which 70% of the data is randomly chosen as the training set and the remaining 30% is used as test data. This method is imprecise and inconsistent when the dataset is limited, and such models are prone to overfitting. However, we employed a stratified k-fold cross-validation strategy, in which the entire data is divided into k segments (k is an integer > 1), with k-1 slices being used to train the model and the remaining slices being used to test the model, as illustrated in Figure 6. The mean scores of the model are calculated by repeating the process k times with k different training and test sets. We picked k = 10, which means there would be ten training cycles with ten separate training and test sets. Cross-validation produces more consistent and accurate ratings, allowing for a more exact assessment of model quality [29].

#### 2.2.4. Two-Class ML Classifier

A binary ML classifier is the final stage in the proposed model, and it is responsible for providing predictions based on test data properties. We explored a number of machine-learning classifier algorithms before deciding that SVM and DNN were the most effective. The next section goes over the two algorithms.

(1) SVM

**Figure 7:**
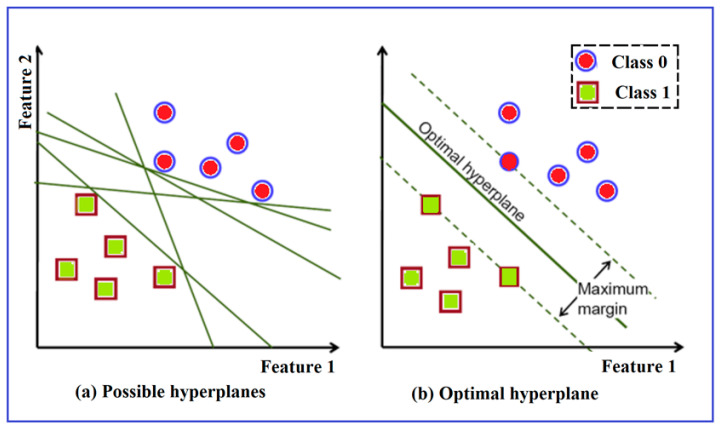
Fitting a hyperplane using SVM; (**a**) plot of a few possible hyperplanes (**b**) Plot of an optimal hyperplane.

SVM is a well-known supervised machine learning algorithm that may be used to classify and predict data. It is a linear classifier that can tell the difference between two classes of linearly separable data with high accuracy. As shown in Figure 7a, many hyperplanes can be created to partition linearly separable data into two groups. The SVM method, on the other hand, finds an ideal hyperplane with a maximum margin between support vectors, as shown in Figure 7b. The support vectors sample data points closer to the hyperplane from both classes that influence the hyperplane’s position and the margin [30,31,32].

Because the SVM classifier requires feature scaling, the data ranges from +1 to −1. The support vector machine produces a real-valued output that is either negative or positive depending on which side of the decision boundary it falls on [27]. The hyperplane distance can be used as a confidence indicator. The more distance between an observation and the plane, the more precise the classification. The SVM classifier’s loss function is hinge loss, which is a type of cost function that determines the cost based on a hyperplane margin. For a target t and prediction p, the loss function *L*(*y*) is defined in Equation (13). Equation (14) gives p, where W is the model weight matrix, b is the bias, and X is the feature inputs. An observation placed directly on the boundary would lose one regardless of whether the ground truth was +1 or −1 [31].

(13) $$L\left(y\right)=\max\left(0,\;1-p.t\right)$$

(14) $$p=w^{T}X+b$$

Although the SVM is a linear model, kernel functions that translate two-dimensional data into higher-dimensional space allow it to handle non-linear datasets [33,34]. The kernel functions are selected based on the dataset’s attributes and must be carefully chosen for high classification accuracy, making it a hyperparameter of the SVM [33]. The most common kernels are linear, polynomial, Gaussian, radial basis, and sigmoid. The SVM algorithm has two other hyperparameters: the regularization coefficient C and gamma factor, γ [34]. C is used to control the model’s overfitting, with typical values ranging from 0.1 to 100. Regularization is higher when C is low, and the model tries to optimize the margin by permitting misclassifications. The γ coefficient decides the curvature of the decision border in non-linear applications. The typical values of γ are within the range of 0.0001 to 10. Low gamma values indicate a broad similarity radius, meaning that each class has many data points. A high Gamma causes the model to overfit by requiring the data points to be substantially close to each other to be deemed the same group. To get high accuracy, C must be tuned for linear classifiers, whereas C and γ must be optimized for non-linear classifiers [34,35].

(2) DNN

**Figure 8:**
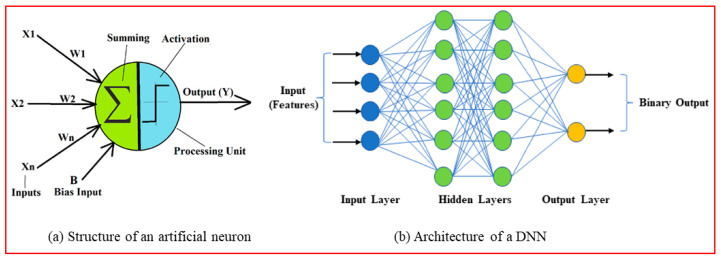
Structure of artificial neuron network [37].

A DNN is a feed-forward artificial neural network (ANN) built using numerous artificial neurons, each of which mimics a biological neuron [36]. As described in Figure 8a, an artificial neuron has N number of inputs (X_i_) to collect the input data and a processing unit with a summing and an activation function to produce an output (Y) [37], using Equation (15), where Wi*Xi is the weighted inputs, B is the bias, and φ is an activation function, yields the output Y. The activation function specifies how the weighted sum of the input is turned into an output. The activation function chosen has a significant impact on the neural network’s capabilities and performance. There are various activation functions to choose from, but the most popular are sigmoid, tanh, and rectified linear unit (ReLU) [37].

(15) $$Y=\varphi (\sum_{i=1}^{N}W_{i}*X_{i}+B)$$

The architecture of a typical DNN network is shown in Figure 8b, which includes an input layer, many hidden layers, and an output layer. The input layer receives the data features, which are subsequently processed by hidden layers to produce an output at the output layer. The learnable parameters of every neural network model are the weights W_i_ and the bias B, which are learned during the model’s training iterations [38]. Tuning a set of DNN hyperparameters is essential to attain optimal performance from these models. The number of neurons, activation function, number of hidden layers, optimizer, learning rate, batch size, and number of epochs are all important hyperparameters. 

Table 2 gives the architecture of the proposed DNN classifier. It consists of an input layer to receive the ten GLCM features, followed by five hidden layers and an output binary classifier layer. Each hidden layer is built using a dense layer, a dropout layer, and a batch normalization layer in cascade. The output dimensions and the number of parameters at each layer are given in Table 2. The dropouts in the dense layers limit the overfitting tendency of the model; similarly, batch normalization layers provide the DNN model with better regularization. 

#### 2.2.5. Metrics for Model Evaluation

The following metrics were used to evaluate the model scores [39,40].

(a) Sensitivity

Sensitivity is also called recall or True positive rate (TPR). It is a ratio of true positives (TP) to the sum of TP and false negatives (FN). A model with high TPR will have few false negatives (FN), which means missing a few positive instances.

(b) Specificity

Specificity is also called true negative ratio (TNR). It is a ratio of true negative (TN) to the sum of TN and false positives (FP). A model with high TNR means that the model is correctly identifying most of the negative results.

(c) Precision

Precision measures the accuracy of a model’s positive prediction. Precision is calculated by dividing the TP by the total number of positive cases (TP + FP) in the test samples.

(d) F1-Score

F1-score is determined using the sensitivity and precision scores of a model, and its value ranges from 0 (min) to 1 (max). F1-score can be calculated using Equation (16).

(16) $$F1=2\;x\;\frac{Precision*\;Sensitivity\;}{Precision+Sensitivity\;}$$

(e) Accuracy

The model’s accuracy is defined as the ratio of correct predictions (TP+TN) to the total number of samples utilized to test the model. Because the default threshold for determining the TP, TN, FP, and FN is 50%, the above three metrics may be approximate.

(f) The area under the receiver operating characteristics (AUROC or AUC)

AUC is a better measure of accuracy since it is a cumulative model score across all classification thresholds [40]. The ROC is a two-dimensional plot with the FPR (complement of TNR) on the horizontal axis and the TPR on the vertical. Area under the ROC curve gives AUC that tells how well the model discriminates the negative and positive classes. 

#### 2.2.6. Implementation of DNN Model

The following hyperparameters were chosen as the best for training the model: activation function: ReLU, dropout: 0.5, momentum = 0.95, epsilon = 0.001, optimizer: Adam, batch size: 32, epochs: 100, and learning rate: 0.001. We used the training data to perform DNN training and plot the model’s accuracy and loss curve. We ran tests on the trained DNN model using the test data, displayed the ROC, and calculated model scores. We utilize Google Drive for data storage and implemented the algorithm in python on Google Colaboratory and a Dell XPS 15 9500 laptop with an Intel(R) Core (TM) i7-10750 H processor running at 2.60 GHz, 2592 MHz, 6 Cores, and 12 Logical Processors as a local computer (US Corporate Headquarters, 1 Dell Way, Round Rock, TX 78664).

## 3. Results

As mentioned earlier, GLCM feature extraction, data splitting, training, and testing of SVM and DNN models are the primary steps involved in the proposed approach. The initial stage GLCM features are extracted from the entire dataset, which consisted of 560 images divided into two categories. The extracted features were saved in an excel file and later used to train and test the models. 

The stratified k-fold CV was used to make the required training and test data with k = 10, providing a training set of size 504 × 11 and test data of size 56 × 11. Feature columns and target column were isolated from these data splits, providing two feature sets (x-train, x-test) and two target arrays (y-train, y-test). The x-train and y-train arrays were used to train the model; the x-test and y-test arrays were used to test the trained model.

The hyperparameter settings used to train the SVM model were: kernel type: polynomial, C = 1, and γ = 1. We carried out training using the training data and then tested the trained SVM model using the test data and recorded the model scores.

**Figure 9:**
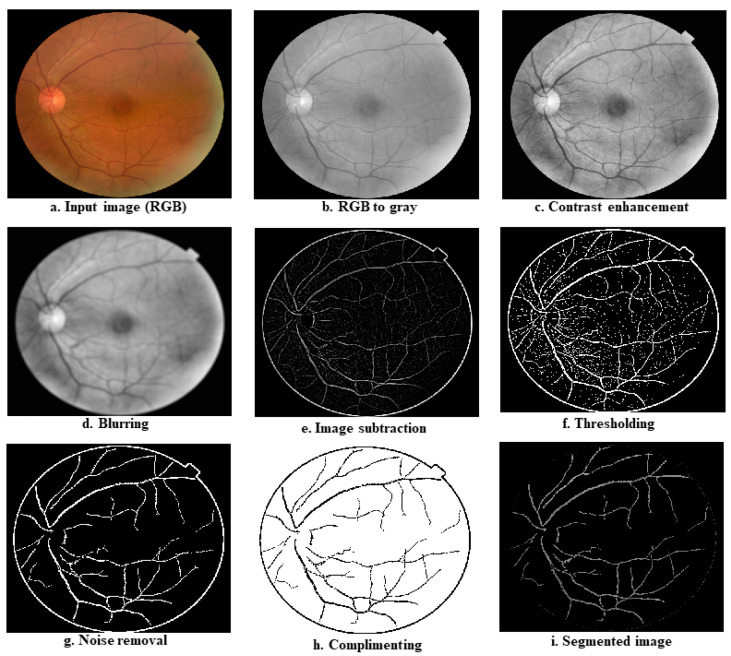
Transformations of input image (normal fundus) at various stages of the segmentation algorithm; (**a**) shows a sample image from the healthy class; (**b**) depicts a gray version of the image; (**c**) provides the image after contrast enhancement operation; (**d**) is the image after averaging operation; (**e**) is the image after subtraction; (**f**) is the image after thresholding operation; (**g**) is the image after noise removal; (**h**) is the image after complementing operation; and (**i**) is the segmented image.

**Figure 10:**
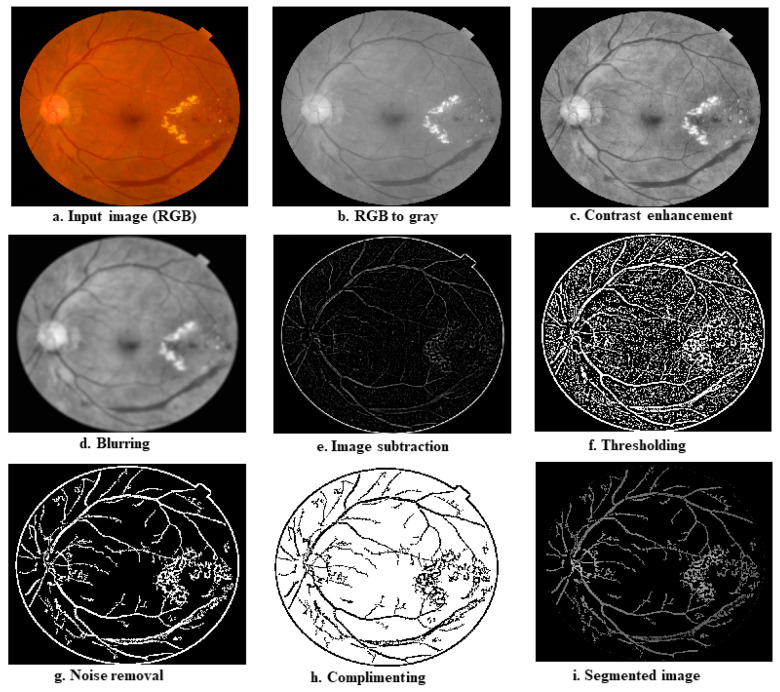
Transformations of input image (DR fundus) at various stages of the segmentation algorithm. (**a**) shows a sample image from the DR class; (**b**) depicts a gray version of the image; (**c**) provides the image after contrast enhancement operation; (**d**) is the image after averaging operation; (**e**) is the image after subtraction; (**f**) is the image after thresholding operation; (**g**) is the image after noise removal; (**h**) is the image after complementing operation; and (**i**) is the segmented image.

As described in the preceding section, we first segmented the entire collection of retinal images in the dataset. Figure 9a shows a sample image from the healthy class; Figure 9b depicts a gray version of the image; Figure 9c provides the image after contrast enhancement operation; Figure 9d is the image after averaging operation; Figure 9e is the image after subtraction; Figure 9f is the image after thresholding operation; Figure 9g is the image after noise removal; Figure 9h is the image after complementing operation; and Figure 9i is the segmented image. Figure 10a–i show a DR image and its transformations at different phases of the segmentation algorithm.

**Figure 11:**
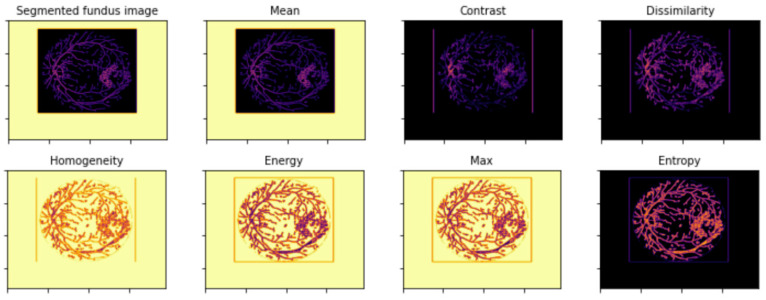
Visualization of GLCM features of a normal fundus sample.

**Figure 12:**
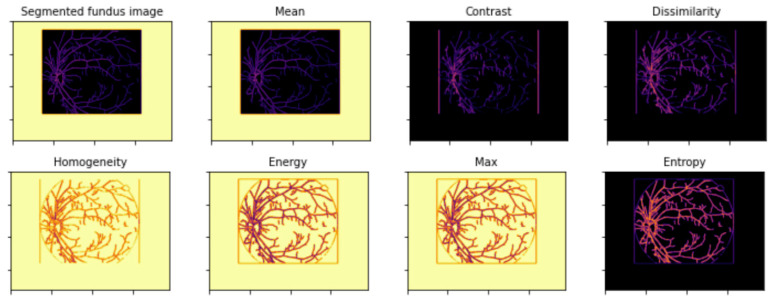
Visualization of GLCM features of a DR fundus sample.

The GLCM algorithm took the segmented images and computed ten GLCM features, as described in Section 2.2.2. Figure 11 and Figure 12 presents visualization of the GLCM features for a normal and a DR sample, respectively.

As described in the preceding section, the next stage was the data splitting, in which the GLCM features are divided into training and test sets. Ten training rounds with ten different training sets and test sets were carried out to cross-validate the model’s score. The performance indicators (mean score with SD) of both models are summarized in Table 3.

**Figure 13:**
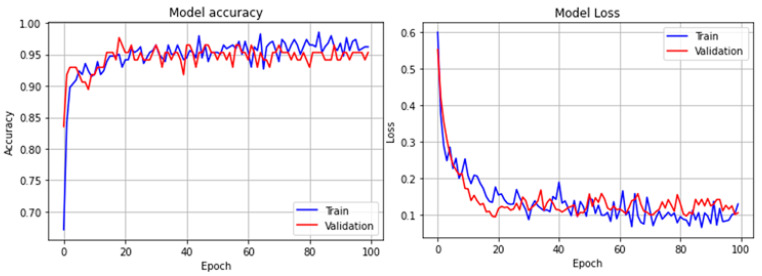
Accuracy and loss plots of DNN model.

The SVM model achieved the following cross-validated scores: accuracy 94.59%, sensitivity 93.65%, specificity 95.13%, precision 90.28%, F1 91.88%, and AUC 97.11%. The model’s accuracy and loss plots during training and validation or testing of the DNN model are shown in Figure 13.

**Figure 14:**
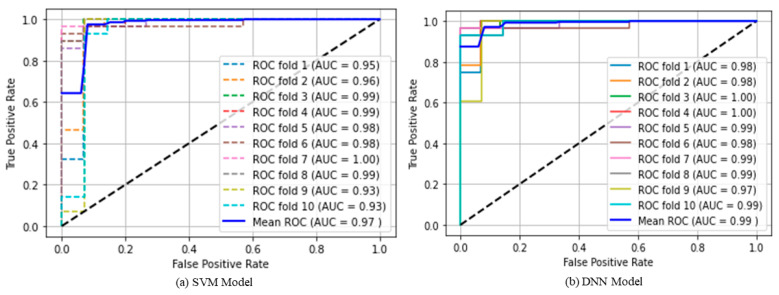
Cross validated scores of SVM and DNN models on test data.

It is evident from the plots that the DNN model began to converge at epoch 40 and reached its maximum convergence at epoch 63. The following scores (mean) were obtained using the DNN model: accuracy 95.77%, sensitivity 95.02%, specificity 95.17%, precision 93.10%, F1-score 93.72%, and mean AUC: 99.15%. Figure 14 shows the ROC plots, mean ROC plot, AUC scores, and mean AUC score of both the models.

## 4. Discussion

DR is a progressive eye disease induced by diabetes that can result in vision loss or blindness if left untreated. Getting checked early is the best way to avoid the problem. A comprehensive dilated eye exam is the best way to diagnose diabetic retinopathy. A eye specialist uses an ophthalmoscope to look for abnormalities such as damage to blood vessels to diagnose DR. Furthermore, fundus images captured by specialized imaging devices support an eye specialist in manually assessing and diagnosing the disease. However, manually administered DR diagnosis has a high degree of subjectivity, is prone to errors, and creates significant delays. 

In this study, we investigated the applicability of ML algorithms for detecting DR using digital fundus images, thereby improving the efficacy of the ongoing DR diagnosis. Using the GLCM features obtained from 560 fundus images, we build two classifier models: the SVM and DNN. We trained the models using 504 images and then carried out tests on 56 images to evaluate the model’s performance. The DNN classifier outperformed the SVM classifier on all the performance metrics and achieved a mean AUC of of 99.15% against 97.11%. DNN models are complex models with many hyperparameters that may be tuned to achieve optimal classification accuracy. Though the DNN model includes 715521 parameters, 711553 of which are trainable, it is less dense than the state-of-the-art ResNet model, which has around 11 million parameters. Contrarily, the SVM is a compact, simple-to-implement model that only needs a few hyperparameters to be adjusted, making it appropriate for usage even on low-power computers. The SVM model performed significantly faster, completing ten rounds of training and tests in 5 s compared to 196 s for the DNN model. It is challenging to draw broad comparisons since, as Table 4 shows, there are differences in the datasets utilized by the previous authors such as the quantity of training, test images and its resolution, techniques adopted, and metrics employed to evaluate the model’s performance.

It is important to note that the dataset we adopted was perfectly balanced (meaning an equal number of normal and DR images), allowing the model to make a unbiased prediction. We evaluated the model’s performance using accuracy, AUC, sensitivity, and specificity. We also considered the training time of the models. Our GLCM-SVM model outperformed the previously published GLCM-SVM model, with an accuracy of 94.59% (vs. 82.35%), AUC of 97.11%, sensitivity of 93.65% (vs. 76.92%), and specificity of 95.13% (vs. 72.58%). Our GLCM-SVM model completed training and cross-validation in 5 s. As we learned, the state-of-the-art model achieved a test accuracy of 96.35% using a ResNet model; the model took 31 min to complete the training. However, the authors did not estimate AUC, sensitivity, or specificity. Our DNN-based DR classifier achieved an accuracy score of 95.77%, an AUC of 99.15%, a sensitivity of 95.02%, and a specificity of 95.17%; all the scores were cross-validated using 10-fold CV. The training of our DNN model was completed in 121 s (about 2 min), nearly 16 times faster than the ResNet model. The suggested DNN model would be appropriate for clinical applications because, in medical environments, prediction scores of 95% or higher are considered acceptable.

We believe that the proposed method would aid an ophthalmologist in developing a speedy and accurate diagnosis because it just takes a few seconds to screen a fundus image. According to us, the proposed models have the following limitations. Although the proposed technique has an AUC of above 99% for detecting DR, it does not provide information regarding the severity of the sickness. Furthermore, for low-income people, fundus imaging is extremely expensive. As a result, we plan to create a low-cost retinal imaging device that combines a lens and a smartphone with an ML algorithm to provide accurate DR classification and grading, allowing patients to conduct retinal screenings at home and seek further referrals as needed.

## 5. Conclusions

This study developed two ML approaches for identifying DR using pre-recorded colored retinal images. The major processes in the offered methodologies are image segmentation, feature extraction, and classification. DR relevant areas were identified using segmentation techniques and then extracted GLCM features from it to develop an SVM and a DNN model. The DNN model excelled both the proposed SVM and the state-of-the-art models on test data. The DNN-based DR classifier takes a few seconds to produce the test result for a given retinal image. With the use of a low-cost fundus image acquisition apparatus that uses a lens and a smartphone, we plan to develop a user-friendly DR classifier for home usage in the future.

## Figures and Tables

**Table 1:** Details of dataset.

Image Dataset	No. of Images	Image Size	Total Images
Kaggle	38 Normal, 47 DR	3046 × 2572	280 Normal 280 DR
DDR	140 Normal	512 × 512	
Mendeley Data	82 Normal	1504 × 1000	
Zenodo	20 Normal, 233 DR	2124 × 2056	

**Table 2:** Configuration of the DNN model.

Layer (Type)	Type of Layer	Output Shape	Number of Parameters
Inpur_1	Input layer	(None, 10)	0
Dense layer_1	Hidden layer_1	(None, 1024)	11,264
Dropout_1		(None, 1024)	0
Batch Normalization layer_1		(None, 1024)	4096
Dense layer_2	Hidden layer_2	(None, 512)	524,800
Dropout_2		(None, 512)	0
Batch Normalization layer_2		(None, 512)	2048
Dense layer_3	Hidden layer_3	(None, 256)	131,328
Dropout_3		(None, 256)	0
Batch Normalization layer_3		(None, 256)	1024
Dense layer_4	Hidden layer_4	(None, 128)	32,896
Dropout_4		(None, 128)	0
Batch Normalization layer_4		(None, 128)	512
Dense layer_5	Hidden layer_5	(None, 64)	8256
Dropout_5		(None, 64)	0
Batch Normalization layer_5		(None, 64)	256
Dense_6	Output layer	(None,1)	65

**Table 3:** Model’s scores on test data.

Model	% Acc.	% Sens.	% Spec	% Prec.	% F1-Score	% AUC
SVM	94.59 +/−0.018	93.65 +/−0.038	95.13 +/−0.016	90.28 +/−0.035	91.88 +/−0.028	97.11 +/−0.023
DNN	95.77 +/−0.017	95.02 +/−0.017	95.17 +/−0.019	93.10 +/−0.037	93.72 +/−0.026	99.15 +/−0.008

**Table 4:** Comparison of the proposed DR classifier with state-of-the-art approaches.

Author	Methods	Number of Images	Training and Test Data	%Acc.	%AUC	%Sens	%Spec
Ahmed Z. et al., 2018 [16]	GLCM, SVM	Training: 27 Test: 17	70: 30 split	82.35	N.A.	76.92	72.58
K. Xu et Al., 2019 [17]	CNN	Test:1000	70: 30 split	94.50	N.A.	N.A.	N.A.
Dhiravidachelvi et al., 2019 [18]	GLCM, KNN.	Test: 100	Fixed test samples	93.0	N.A.	95.70	90.56
Elveny M. et al., 2020 [19]	GLCM, Prob. NN.	Normal: 470, DR: 555	80: 20 split	86.80	N.A.	N.A.	N.A.
Ramzi et al., 2021 [20]	LBP, CNN-ResNet	Training: 3662 Test: 1928	Fixed test samples	96.35	N.A.	N.A.	N.A.
Proposed Method -1	GLCM, SVM	Normal: 280, DR: 280	10 Fold CV	94.59	97.11	93.65	95.13
Proposed Method -2	GLCM. DNN	Normal: 280, DR: 280	10 Fold CV	95.77	99.15	95.02	95.17

## Data Availability

The data analyzed during the current study and the extracted features are available from the corresponding author on reasonable request. Due to the size of the image dataset, the data are not yet open to the public.
